# The impact of social well-being on students’ academic motivation and academic achievement: a case study from Iran

**DOI:** 10.1186/s12909-025-08109-3

**Published:** 2025-11-14

**Authors:** Hamed Rahimi, Hooman Etedali, Mohammad Latifhadad

**Affiliations:** 1 School of Medicine, Dezful University of Medical Sciences, Dezful, Iran; 2 Student Research Committee, Dezful University of Medical Sciences, Dezful, Iran

**Keywords:** Academic motivation, Social well-being, Academic achievement, University, Student

## Abstract

**Background:**

Students’ social well-being can significantly enhance their performance in various areas, including academic motivation and achievement. Therefore, this study was designed and conducted to investigate social well-being, academic motivation, and academic achievement among students at Dezful University of Medical Sciences (DUMS).

**Methods:**

The present research was a correlational, descriptive-analytic study conducted as a cross-sectional design. The research sample consisted of 261 students from DUMS. Data were collected using a demographic questionnaire, Keyes’ Social Well-being Scale (SWBS), and Vallerand’s Academic Motivation Scale (AMS). Data were analyzed at the 0.05 significance level using SPSS version 26.

**Results:**

According to the results, the average scores of students’ academic achievement, academic motivation, and social well-being were 17.12 ± 1.23, 127.16 ± 25.33, and 76.61 ± 11.35, respectively. Social well-being had a significant positive correlation with academic motivation (*r* = 0.234), but no significant correlation was found between social well-being and academic achievement (*r* = 0.077). Additionally, no significant correlation was observed between students’ academic motivation and achievement (*r* = 0.084). Results also revealed that social well-being is associated with 28.5% and 1.11% of the variance in academic motivation (ADJ.R^2^ = 0.285) and achievement (ADJ.R^2^ = 0.111), respectively.

**Conclusion:**

Social well-being, particularly the components of participation, solidarity, and social acceptance, is a relevant factor in students’ academic motivation. Thus, considering the moderate levels of social well-being and academic motivation among students, the officials of DUMS, through proper and principled planning in providing students’ social well-being, can contribute to enhancing students’ academic motivation, which may ultimately lead to educational achievement.

**Supplementary Information:**

The online version contains supplementary material available at 10.1186/s12909-025-08109-3.

## Background

Universities, as the highest centers of thinking and knowledge production in society, play a fundamental role with the presence and activities of thinkers, researchers, scholars, and students [1]. Globalization, internationalization, and the information technology revolution have forced universities to participate in competitive education, research, and entrepreneurship markets. Universities must quickly adapt to this changing environment to maintain and enhance their position [2, 3]. Universities can strive to increase efficiency and optimize the use of existing resources through careful planning in educational, research, and human resource training affairs [4]. A crucial topic in educational systems is academic motivation and achievement to the extent that one of the primary concerns of professors, university administrators, and families is students’ academic achievement and preventing academic failure [5, 6]. Attention to students’ academic achievement and the factors influencing it is a necessary strategy for improving education. While academic achievement is broadly correlated with general intelligence, other concepts, such as motivation, are also significant [7, 8].

Among students, academic motivation, which reflects a willingness to strive for achievement and is a significant predictor of academic success, is of particular importance [7, 9]. In general, academic motivation encompasses the drives, needs, and factors that cause an individual to attend educational institutions and obtain academic degrees [10]. Motivated students have a desire to complete assignments, achieve their objectives, and perform at a certain level, which eventually results in learning success and academic achievement [9]. Highly motivated individuals can complete difficult tasks. Their expectations of failure or success led them to pursue success or avoid failure. Motivation is a crucial feature that influences all human activities [7, 11]. Thus, the issue of increasing academic motivation and achievement is of great importance and concern for medical education managers in various countries [12, 13]. students’ social well-being is one of the factors that can both affect their performance in various fields, including academic motivation and achievement, and be affected by them [14]. An individual’s social well-being refers to the quality of their relationships with family, friends, and social groups. The social well-being index is a part of individual well-being that shows how satisfied or dissatisfied a person is with their life and social environment. In short, social well-being encompasses an individual’s internal responses (emotions, thoughts, and behaviors) [15]. Therefore, enhancing students’ academic motivation and achievement requires modifications in the factors affecting them, including social well-being [16].

In this context, the self-determination theory (SDT) categorizes individual motivation into three main types: intrinsic, extrinsic, and amotivation [17]. According to SDT, humans naturally tend toward dynamism and effectiveness to satisfy the needs of autonomy, competence, and relatedness. Moreover, these three needs are essential for growth, perfection, and overall well-being [18]. The SDT states that fulfilling these needs can boost a person’s motivation through specific motivational processes, and that increased motivation subsequently provides a foundation for promoting social well-being [19]. On the other hand, Keyes’ model suggests that social well-being includes Social actualization, integration, contribution, coherence, and acceptance [20]. In line with Keyes’ framework, social well-being is considered a multidimensional construct and a vital factor in mental health and overall quality of life at both individual and social levels. It has the potential to enhance individuals’ basic needs (e.g., autonomy, competence, and social connection) by providing opportunities and supportive environments [21]. Within this framework, social well-being is not just a direct result of these basic needs but also an important factor in their reinforcement and maintenance. In other words, healthy, supportive, and balanced social environments can strengthen these needs by fostering a sense of belonging, social trust, and acceptance [22]. Therefore, given the positive link between motivation and well-being [23], the interaction between these two models implies that social well-being may act not only as a consequence of basic needs such as autonomy, competence, and relatedness but also as a catalyst and sustainment factor for these needs, creating an ongoing, dynamic cycle among well-being, motivation, and fundamental needs within both individual and social contexts.

Individuals with higher levels of social well-being are more successfully able to cope with the challenges arising from fulfilling social roles. They exhibit greater stability and coherence and have a higher capacity for participation in collective activities. Thus, social well-being serves as a tool for preventing various forms of deviance, and if social well-being decreases or is absent, irreparable complications and problems will arise [24]. However, when students start college, they find themselves in a very different setting from their personal and social lives. Students are more likely to experience stress and harm during their academic careers for a variety of reasons, including being away from family, living in dormitories, entering a larger and more stressful environment, adapting to diverse cultures and people, financial difficulties and insufficient income, the type of activities and roles they undertake, and the numerous expectations placed on them by others and the responsibilities they assign themselves. Therefore, environmental change and the challenges faced during the academic career may compromise one’s social well-being [25]. As a result, students with positive social well-being strive to enhance their academic performance to prevent anxiety arising from social rejection by social groups [14]. Strengthening students’ social well-being enables them to become resilient to problems, adapt to changing life conditions and evolving technology, and play a constructive role in society [26].

Therefore, as students are the vanguard of science, knowledge, and development in all societies and play a crucial role in a nation’s future, paying attention to their individual and social well-being, as well as their academic motivation and achievement, is of paramount importance [15, 27]. Despite their particular psychosocial difficulties, medical sciences students, particularly in high-stress and collectivist environments such as Iran, have hardly ever been the subject of research on the relationship between social well-being and academic motivation and achievement [6, 28]. Recent data indicates that the COVID-19 pandemic’s stressors are making mental health crises among medical sciences students around the world worse [29, 30]. However, underlying factors like the pressure on Iran’s healthcare system and resource shortages caused by sanctions and a rise in elite migration create unique patterns of vulnerability for Iranian medical sciences students [31, 32]. Where students’ academic performance and motivation may be particularly impacted by sociopolitical issues such as collectivist norms, the digital gap, and economic sanctions [33]. The objective of this study is to address this gap by investigating the relationship between academic achievement, academic motivation, and social well-being of medical sciences students, which is influenced by Iran’s unique cultural and economic context. The findings of this study may provide new insights into the neglected relationship between higher education and social well-being. Additionally, it could provide policymakers with the evidence they need to adopt creative educational strategies and practical plans. Therefore, it is essential to conduct further research in this area. Accordingly, the current study, using Keyes’ model and SDT, investigates the role of social well-being in students’ academic motivation and achievement at Dezful University of Medical Sciences (DUMS), aiming to address and reduce the existing knowledge gap.

## Methods

### Study setting and sample

The current study used a cross-sectional design and was correlational, descriptive, and analytical. The research population included all students enrolled at DUMS during the 2023–2024 academic year. Considering a significance level of 0.05 and a power of 80% to detect a minimum correlation coefficient of 0.2, the required sample size was calculated as follows [34]:

$$\:n={\left(\frac{{z}_{\alpha\:}+{z}_{\beta\:}}{0.5*\text{ln}\left(\frac{1+r}{1-r}\right)}\right)}^{2}+3={\left(\frac{1.96+1.28}{0.5*\text{ln}\left(\frac{1+0.2}{1-0.2}\right)}\right)}^{2}+3=256+3=259$$

Consequently, the minimum sample size for this study was calculated to be 259 medical students at DUMS. Considering a 10% dropout rate in the analyzable data, the sample size was 286.

**Fig. 1:**
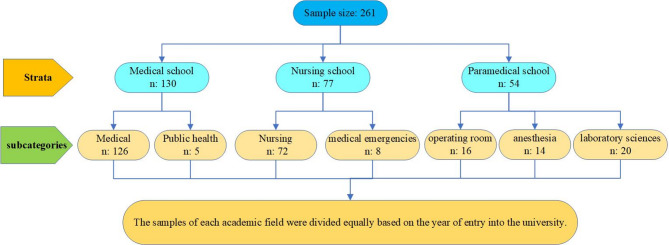
Sample selection process

A multistage stratified random sampling proportional to size was employed. In this method, due to the heterogeneity of the research population, the population was divided into different strata in such a way that individuals within each stratum were homogeneous and similar. In this study, each of the schools (medical, paramedical, and nursing) was considered a separate stratum, and then within each school, academic fields (medical, public health, nursing, medical emergencies, operating room, anesthesia, and laboratory sciences) were selected as subcategories. This classification was based on differences in educational, professional, future roles, levels of stress, and cultures within each faculty. These differences may influence their attitudes, behaviors, and needs. Furthermore, in this method, to ensure that the number of samples selected from each stratum and subcategory was proportional to its population proportion, the proportional allocation was used to determine the sample size for each stratum and subcategory. Subsequently, the required number of individuals from each subcategory was selected through simple random sampling. Finally, the samples of each academic field were divided equally based on the year of entry into the university (Fig. 1). Sampling within each subcategory continued until the predetermined ratio was achieved.

Inclusion criteria included completing at least two academic semesters, consent to participate in the study, and enrollment at DUMS. Exclusion criteria were participant unwillingness to continue participation in the research and failure to complete the questionnaire. The study was conducted after obtaining approval from the Ethics Committee of DUMS.

### Tools and data collection

Data collection tools included a demographic questionnaire, Keyes’ Social Well-being Scale (SWBS), Vallerand’s Academic Motivation Scale (AMS), and academic achievement (measured via students’ Grade Point Average (GPA)). Keyes’ SWBS consists of 28 items across 5 subscales: Social actualization, social integration, social contribution, social coherence, and social acceptance. Items are scored on a five-point Likert scale. Accordingly, the minimum and maximum scores are 28 and 140, respectively. Questions 1–9, 11, 14, and 21 are scored inversely [35]. Babapour et al. used internal consistency to assess the reliability of this questionnaire and reported a coefficient of 0.78, indicating a satisfactory level of reliability of this tool [36]. Vallerand’s AMS consists of 28 items across 7 subscales: Intrinsic motivation (to know, to accomplish things, to experience stimulation), Extrinsic motivation (Identified regulation, Introjected regulation, External regulation), and Demotivation (Lack of motivation). Responses are provided on a seven-point Likert scale (not at all, very low, low, moderate, high, very high, and absolutely). The minimum and maximum possible scores are 28 and 196, respectively. The reliability and validity of the scale have been confirmed in numerous studies [14, 37, 38]. To ensure the accuracy of self-reported GPA data and reduce self-report bias, this data was matched with official data recorded at the university.

Therefore, data were collected after obtaining approval from the Ethics Committee and the Vice-Chancellor for Research at DUMS. During data collection, participants were provided with explanations of the research objectives, reminded to keep the information confidential, and the voluntary nature of their participation. Written and verbal informed consent was obtained from all participants. To guarantee ethical concerns, the study goals were communicated to the students. After assuring them of the confidentiality of their information, participants voluntarily agreed to participate in the study.

Ultimately, 261 responses were collected and analyzed, achieving a 91.25% response rate. This exceeded our initial target (259 participants) and ensured adequate statistical power despite minor attrition. According to the findings, most participants were 18–22-year-old students (59.4%). The majority of the participants were male (63.6%). Single students constituted 91.6% of the sample. Approximately half of the participants were medical students (49.8%). In terms of academic level, most were bachelor’s degree students (49.8%), and in terms of field of study, medical students were the largest group (47.9%). The participants’ average age was 22.51 ± 2.39 years.

### Data analysis

For data analysis, descriptive statistics, including frequency, percentage, average, and Standard Deviation (SD), were employed to describe the data in this study. After examining data normality using the Shapiro-Wilk test, Mann-Whitney U, Kruskal-Wallis, Spearman’s correlation coefficient, and multiple regression analysis were conducted to analyze the data using SPSS version 26 at a significance level of 0.05.

To examine collinearity in regression models, the variance inflation factor (VIF) and tolerance values were calculated. Tolerance and VIF are reciprocals of each other. So that a VIF greater than 10 and a tolerance smaller than 0.1 indicate a problem of multicollinearity [39].

## Results

While academic motivation (127.16/196) and social well-being (76.61/140) were in the moderate range, the average academic achievement score (17.12/20) indicated a high-performance level (Table 1). The findings revealed a statistically significant difference in students’ social well-being based on academic criteria (school, academic level, and field of study) (*P* < 0.05). However, age, gender, and marital status did not significantly differ in social well-being (*P* > 0.05). Gender and age differences in academic motivation were significant; younger students (18–22 years old) and females were more motivated (*P* < 0.05). Marital status, school, academic level, and field of study did not show any significant relationship (*P* > 0.05). There were significant differences in academic achievement by gender, school, academic level, and field of study; bachelor’s students, anesthesia students, paramedical school students, and female students had better academic achievement (*P* < 0.001). Age and marital status did not significantly differ (*P* > 0.05). The Kruskal-Wallis test (for age, school, academic level, and field of study) and the Mann-Whitney test (for gender and marital status) were used in the statistical analyses (See additional file).

**Table 1:** Descriptive statistics of the variables of age, GPA, AMS, and SWBS of students

Variables			Average	Standard deviation	Min	Max
Social well-being	Social actualization		17.87	5.7	8	36
	Social integration		16.27	4.1	8	29
	Social contribution		19.43	3	8	25
	Social coherence		12.75	2.2	7	18
	Social acceptance		10.28	2.1	4	15
	Total		76.61	11.35	43	108
Academic motivation	Intrinsic motivation	To know	21.07	5.2	7	28
		To accomplish things	18.9	5.5	4	28
		To experience stimulation	21.27	5.2	5	28
	Extrinsic motivation	Identified regulation	16.78	3.8	6	25
		Introjected regulation	16.95	5.6	4	28
		External regulation	19.48	5.1	4	28
	Demotivation	Lack of motivation	12.71	4.22	4	25
	Total		127.16	25.33	48	184
Academic achievement			17.12	1.23	13	19.93

The Spearman’s correlation coefficient showed that students’ academic motivation had a weak, positive, and significant relationship with social well-being and its two components (social coherence and social acceptance). Also, academic motivation had a modest, positive, and significant relationship with the social contribution component. However, social well-being and its components did not show a significant relationship with academic achievement. Furthermore, the analyses demonstrated that students’ academic motivation was not significantly correlated with their academic achievement (Table 2).

**Table 2:** Correlation coefficient of social well-being and its components, academic motivation, and academic achievement of DUMS

Variables	1	2	3	4	5	6	7	8
1. Social well-being	1							
2. Social actualization	0.819^*^ (0.76 − 0.85)	1						
3. Social integration	0.634^*^ (0.54 − 0.71)	0.421^*^ (0.30–0.52)	1					
4. Social contribution	0.415^*^ (0.29 − 0.51)	0.069 (−0.6 – 0.19)	−0.001 (−0.14 – 0.12)	1				
5. Social coherence	0.658^*^ (0.57 − 0.73)	0.465^*^ (0.36–0.57)	0.233^*^ (0.11–0.35)	0.308^*^ (0.18 − 0.42)	1			
6. Social acceptance	0.471^*^ (0.36 − 0.57)	0.156^**^ (0.03–0.27)	0.161^*^ (0.04 − 0.28)	0.376^*^ (0.26 − 0.48)	0.368^*^ (0.25 − 0.48)	1		
7. Academic motivation	0.234^*^ (0.11 − 0.34)	0.114 (−0.09 – 0.23)	−0.049 (−0.18 − 0.07)	0.455^*^ (0.35 − 0.56)	0.239^*^ (0.11 − 0.37)	0.205^*^ (0.08 − 0.33)	1	
8. Academic achievement	0.077 (−0.04 − 0.2)	0.06 (−0.06 – 0.18)	0.085 (−0.04 − 0.21)	−0.01 (−0.12 − 0.11)	0.045 (−0.07 − 0.18)	0.116 (−0.02 − 0.25)	0.084 (−0.03 − 0.2)	1

Values in parentheses represent the 95% confidence interval for each correlation coefficient

Correlation coefficients (Spearman’s r) were calculated, with ^*^ indicating *P* < 0.01 and ^**^ indicating *P* < 0.05

The Spearman’s correlation coefficient showed that there was a significant and positive correlation between social well-being and the five components of academic motivation (to know, to accomplish things, to experience stimulation, identified regulation, and injectable regulation), while there was a significant negative correlation with the lack of motivation (*P* < 0.01). Correlation analyses between the components of the two variables of academic motivation and social well-being indicate that the components of social contribution and social coherence have a positive and significant correlation with all components of academic motivation, except for the lack of motivation (*P* < 0.01). In contrast, the component of social integration showed a negative and significant relationship only with the lack of motivation (*P* < 0.01). Overall, the Lack of motivation negatively and significantly correlated with every component of social well-being, except for social actualization (*P* < 0.05) (Table 3).

**Table 3:** Correlation between components of social well-being and academic motivation of DUMS students

Social well-being Academic motivation		Actualization	Integration	Contribution	Coherence	Acceptance	Total
Intrinsic motivation	To know	− 0.001 (−0.13 − 0.12)	0.032 (−0.1 − 0.15)	0.499^*^ (0.4 − 0.6)	0.235^*^ (0.10 − 0.35)	0.239^*^ (0.12 − 0.35)	0.233^*^ (0.1 − 0.34)
	To accomplish things	0.22^*^ (0.09 − 0.34)	0.085 (−0.04 − 0.21)	0.441^*^ (0.32 − 0.55)	0.297^*^ (0.18 − 0.41)	0.289^*^ (0.16 − 0.39)	0.359^*^ (0.24 − 0.47)
	To experience stimulation	0.075 (−0.06 − 0.2)	− 0.017 (−0.15 − 0.10)	0.458^*^ (0.34 − 0.56)	0.234^*^ (0.10 − 0.35)	0.273^*^ (0.15 − 0.39)	0.24^*^ (0.11 − 0.36)
Extrinsic motivation	Identified regulation	0.133^*^ (0.01 − 0.25)	− 0.033 (−0.16 − 0.09)	0.383^*^ (0.26 − 0.50)	0.255^*^ (0.14 − 0.37)	0.17^*^ (0.05 − 0.28)	0.235^*^ (0.11 − 0.35)
	Introjected regulation	0.161^*^ (0.02 − 0.29)	− 0.08 (−0.2 − 0.05)	0.322^*^ (0.20 − 0.43)	0.182^*^ (0.06 − 0.31)	0.073 (−0.05 − 0.19)	0.174^*^ (0.05 − 0.3)
	External regulation	0.045 (−0.09 − 0.17)	− 0.119 (−0.24 − 0.002)	0.316^*^ (0.19 − 0.43)	0.127^*^ (−0.002 − 0.25)	0.104 (−0.02 − 0.22)	0.097 (−0.03 − 0.21)
Demotivation	Lack of motivation	− 0.001 (−0.14 − 0.13)	− 0.144^*^ (−0.26 - − 0.02)	− 0.219^*^ (−0.33 - − 0.09)	− 0.136^**^ (−0.25 - − 0.006)	− 0.213^*^ (−0.32 - − 0.09)	− 0.168^*^ (−0.29 - − 0.03)

Values in parentheses represent the 95% confidence interval for each correlation coefficient

Correlation coefficients (Spearman’s r) were calculated, with ^*^ indicating *P* < 0.01 and ^**^ indicating *P* < 0.05

The impact of social well-being factors on students’ academic motivation and achievement was examined using a multiple regression analysis. Given that academic motivation was significantly correlated with the variables of gender, age, and number of semesters completed, and that academic achievement was significantly correlated with the variables of School and gender, multiple regression analysis was employed to adjust for the effects of these variables. Considering the significant *p*-value (*P* < 0.001), the multiple regression models revealed a good fit, indicating that the independent factors significantly explained students’ academic motivation and achievement. Among the predictor variables, social contribution had a significant explanatory power for students’ academic motivation, according to the simultaneous multiple regression analysis (*P* < 0.05). Among the predictor variables, social contribution exhibited significant predictive power for the variable of students’ academic achievement (*P* < 0.05). Analyses revealed that the predictor variables collectively explained 28.5% and 11.1% of the variance in academic motivation (ADJ.R^2^ = 0.285) and achievement (ADJ.R^2^ = 0.111), respectively. Standardized regression coefficients (β) indicate that, controlling for confounding variables, a one-standard-deviation increase in the social contribution score leads to a 0.38-standard-deviation increase in the students’ academic motivation score, and vice versa. Additionally, standardized regression coefficients (β) indicate that controlling for confounding variables, a one standard deviation increase in the social acceptance score will lead to a 0.136 standard deviation increase in students’ academic achievement, and vice versa (Table 4). Examining the collinearity between independent variables using VIF and Tolerance indices showed that VIF values were close to 1 (range 1.06 to 1.58) and Tolerance values were less than 1 (range 0.65 to 0.95), indicating the absence of problematic collinearity in both models.

**Table 4:** Multiple regression analysis to predict students’ academic motivation and achievement through social well-being

Indicators Variables	B	SE	β	t	*P* ^*^	*R*	*R* ^2^	ADJ.*R*^2^	*P*
Academic motivation*									
Constant	131.7	18.9		6.97	0.000	0.554	0.307	0.285	0.000
Actualization	0.48	0.29	0.107	1.63	0.104				
Integration	− 0.64	0.36	− 0.105	−1.77	0.077				
Contribution	3.26	0.5	0.38	6.5	0.000				
Coherence	0.64	0.77	0.05	0.83	0.406				
Acceptance	− 0.08	0.74	0.007	−0.11	0.914				
Academic achievement**									
Constant	16.86	0.64		26.34	0.000	0.368	0.135	0.111	0.000
Actualization	0.017	0.016	0.081	1.12	0.263				
Integration	−0.001	0.02	−0.002	−0.032	0.97				
Contribution	− 0.028	0.026	− 0.07	−1.1	0.27				
Coherence	−0.011	0.041	−0.02	− 0.268	0.79				
Acceptance	0.08	0.039	0.136	2.02	0.044				

*Regression coefficients were adjusted for age, gender, and Semesters passed

**Regression coefficients were adjusted for gender and School

## Discussion

This study was designed and conducted to investigate the role of social well-being in students’ academic motivation and achievement at DUMS. The participants’ average age was 22.51 ± 2.39 years, their average social well-being score was 76.61 ± 11.35, their average academic motivation score was 127.16 ± 25.33, and their average academic achievement score was 17.12 ± 1.23.

Data analysis revealed that the average social well-being score among students at DUMS was moderate. Ajam’s study at Gonabad University of Medical Sciences reported an average social well-being score of 96.51 ± 12.46 for students, which was higher than the present study [14]. Furthermore, data analysis indicated that the average academic motivation score among students at DUMS was moderately high (64.8%). Ajam’s study at Gonabad University of Medical Sciences reported an average academic motivation score of 96.05 ± 21.35 for students, which was lower than the present study [14].

Furthermore, the findings revealed significant differences in students’ social well-being based on School, academic level, and field of study. Results demonstrated that public health and medical students had the highest and lowest levels of social well-being, respectively. However, in Bahmaei et al.’s study, laboratory sciences and health information technology students had the highest and lowest levels of social well-being, respectively [6]. The studies conducted by Bahmaei et al. and Yazdanpanah and Nikvarz found significant differences in social well-being among different academic fields of study [6, 26], which aligns with the findings of the current study. However, the results of Ajam’s study showed no significant differences in social well-being among students in different fields of study [14], which is inconsistent with the current study. Furthermore, Bahmaei et al. found a significant difference in social well-being among different academic levels [6], which is consistent with the present study. On the contrary, Rezadoust et al. did not find any significant difference between academic level and social well-being [27], which is inconsistent with the current study. Modiri et al.’s study also revealed no significant correlation between academic level and social well-being [24], which is not in line with the present study. In explaining this finding, it can be said that academic level and field of study are significantly related to social well-being, but which academic level or field of study has higher social well-being varies across different societies and depends on other factors, such as environmental, social, cultural issues, the educational system’s characteristics, and other variables that require further investigation and research.

The findings indicated that female students had higher academic motivation than male students; this difference was statistically significant. However, Bahmaei et al.’s study revealed that although male students generally exhibited higher academic motivation, this difference was not statistically significant [6]. In Yousefi et al.’s study, male students demonstrated a higher motivation for competition and task completion compared to female students [16], which is not consistent with the present study. Fata et al.’s study showed no significant correlation between gender and academic achievement among students [40], which is not consistent with the present research. Furthermore, the analyses revealed a significant correlation between students’ academic motivation and their age, with younger students exhibiting higher levels of academic motivation. However, Bahmaei et al.’s study found no significant correlation between academic motivation and its components and age, except for demotivation [6], which showed a positive correlation with age. Thus, it can be said that academic motivation is significantly correlated with gender and age; however, which group has higher academic motivation varies across different societies and depends on other factors, such as the level of needs, environmental, social, and cultural issues, the educational system’s characteristics, career concerns, future job security, and other variables that require further investigation and research.

Overall, the analyses revealed a weak, positive, and significant correlation between academic motivation and social well-being (*r* = 0.234, *p* < 0.01). In other words, the higher the students’ social well-being, the higher their academic motivation will likely be. Moreover, the weak and negative correlation between demotivation and social well-being (*r* = −0.168, *p* < 0.01) indicates that low social well-being may contribute to increased demotivation. We can understand that despite a student having low social well-being, there may be other factors that increase his or her academic motivation and vice versa. Previous studies have also emphasized the positive correlation between academic motivation and social well-being [6, 41], but this correlation was at a moderate level. This may reflect differences in the characteristics of the study population (including economic and cultural) or methodological differences. Therefore, it is suggested that further research in the future should examine the factors affecting the relationship between social well-being and academic motivation, to determine which factors have a positive effect on a student’s academic motivation, even in the case of low social well-being.

The observed correlation between social well-being and academic motivation (*r* = 0.234), although small in magnitude, is significant within the complex and multifactorial nature of academic outcomes. In such contexts where numerous individual, social, and institutional factors are at play, it is valuable to identify any meaningful and modifiable associated factors. This finding suggests that social well-being is a relevant piece of the motivational puzzle. Even a small effect can have practical implications when considered at the population level, as scalable interventions targeting social well-being could potentially yield meaningful cumulative benefits for student engagement. It is worth noting that this correlation is small and insufficient to design appropriate interventions alone; other effective factors should also be examined.

Results demonstrated that social well-being and academic motivation had no significant correlation with academic achievement (measured via GPA). Additionally, according to Bahmaei et al.’s study, social well-being and academic motivation had no significant correlation with students’ academic achievement [6]. However, Ajam’s study demonstrated a significant positive correlation among all components of social well-being and academic achievement [14]. Baby et al.’s study also revealed a significant positive correlation between social well-being and academic performance, indicating that as students’ social well-being increased, so did their academic performance [42]. Yousefi et al. also argued that motivational factors significantly impact academic achievement, and higher motivation is associated with higher average grades among medical students [16].

Discrepancies in findings across various studies may be attributed to variations in personal characteristics, academic environments, and cultural, social, and family circumstances of the study participants. Amrai et al. attributed the weak correlation between academic achievement and academic motivation and its components to the inaccuracy in completing questionnaires and a lack of honesty in reporting students’ GPAs [43]. Researchers have identified factors such as inadequate educational and welfare environments, uncertain career prospects, individual characteristics, and unmet expectations from the educational environment as contributors to declined academic motivation among students, even those with satisfactory academic achievement [6]. In essence, despite having satisfactory academic achievement, students’ academic motivation may decline due to environmental, social, economic, political, and other factors. Additionally, students’ academic achievement is largely related to their intelligence and talent. On the other hand, students’ academic motivation may not be solely focused on academic achievement and getting a high GPA; instead, their academic motivation may be driven by the desire for social and family status, a suitable career, etc. Findings suggest a positive correlation between social well-being and academic motivation.

Based on multiple regression analysis, social well-being has a significant relationship with students’ academic achievement, and the components of social well-being account for 11.1% of the variance in students’ academic achievement. Furthermore, social well-being contributed to students’ academic motivation, and the components of social well-being account for 28.5% of the variance in students’ academic motivation. Ajam’s study also revealed that components of social well-being account for 46.5% of the variance in students’ academic motivation [14]. Baby et al.’s study also demonstrated that social well-being and the social cohesion and social participation components were significant positive predictors of students’ academic performance [42]. Evidence also supports the notion that social well-being is a powerful variable influencing students’ physical and mental health, academic performance, and personal growth [44, 45]. This indicates that additional factors are also effective in determining the academic achievement and motivation of students. As evidence has shown, various factors, including demographic, entrance scores, family socioeconomic status, family education level, family support, welfare facilities, student e-learning activities, study skills, physical and mental health status, faculty management style, and university environment, have an impact on academic achievement [46–48]. Furthermore, the structural pattern of student motivation can be effectively explained by a combination of educational, personal, sociocultural, economic, and environmental factors [49]. Of course, it should be noted that due to the type of study (cross-sectional), the present results only highlighted the correlation between social well-being, academic motivation, and students’ academic achievement. Therefore, to further investigate and discover the cause-and-effect relationships between them, it is necessary to conduct studies with a longitudinal or experimental design. Nevertheless, examining the correlations found in this study can serve as a basis for future research with long-term and experimental designs, in order to provide stronger inferences about causal relationships.

It is worth noting that, although bivariate correlation analysis did not reveal a significant relationship between social well-being and academic achievement (*r* = 0.077), the regression model adjusted for gender and school showed that social well-being is associated with 11.1% of the variance in academic achievement (ADJ.R2 = 0.111). This suggests that the correlation between these two variables is not strong enough to be significant at the overall level without controlling for other variables, but in the regression model, it plays a meaningful role in predicting academic achievement. This difference may stem from the different nature of these analyses; while correlation only assesses the linear relationship between two variables, multivariate regression can identify more complex relationships and moderating effects by accounting for other variables [50, 51] (such as gender and school in this model). In other words, the impact of social well-being on academic achievement might vary across gender groups or schools, or this relationship could become apparent only when considering moderating variables. This finding highlights the importance of considering contextual factors and using multivariate analyses, as true relationships may only emerge under specific circumstances or in conjunction with other factors.

Evidence suggests that students’ participation and engagement in the social system have an impact on the level of student cohesion, ultimately leading to increased academic motivation and academic performance [41, 52]. Research has shown that individuals are most significantly influenced by social factors and social cohesion [41, 53]. Although the findings of this study demonstrate that the academic achievement of medical students at DUMS is minimally related to social well-being and its components, there is a significant correlation between academic motivation and social well-being and its components. Yousefi et al. also note that, as the success of medical students is closely related to social well-being, their motivating factors should receive greater attention [16]. Ajam also emphasizes that since social well-being can correlate with an individual’s academic motivation, students with higher levels of social well-being are more motivated and enthusiastic about their studies. Therefore, this correlation between social well-being and academic motivation suggests that interventions designed to strengthen students’ social well-being could be a promising area for future research to explore their potential effects on student dynamism, vitality, and effort. Such vitality and dynamism will subsequently pave the way for students to improve their academic achievement [14]. Consequently, the association between social well-being and academic motivation suggests that attention to social well-being could be a valuable consideration in efforts aimed at understanding and fostering student motivation. Based on the study results, it is recommended that policymakers and decision-makers in medical education systems pay special attention to developing and implementing educational and cultural programs, such as life skills training, interpersonal relationships, and social relationships, to strengthen and improve students’ social well-being and motivation for achievement.

### Practical and research recommendations

Universities can raise students’ social well-being and academic motivation by implementing several key strategies. The study’s findings allow for the classification and recommendation of practical strategies into two main categories: interventions that enhance social well-being and targeted motivational interventions. Implementing social integration programs such as creating student support groups in universities, holding workshops on communication skills and emotional intelligence, implementing programs that strengthen social skills, healthy relationships, and a sense of belonging for students, and developing digital platforms to facilitate students’ social interactions, are some interventions that can be recommended to enhance students’ social well-being. Also, considering the relationship between academic motivation and social well-being, designing motivational programs based on a social approach, such as pair mentoring systems between students with high and low social well-being; designing psychological support packages for students with low motivation; integrating social components into curricula, such as cooperative learning; and creating motivation to participate in charity, social projects, and community service activities are recommended as targeted motivational interventions. At the educational policy level, it is also recommended to allocate optional courses focusing on socio-academic skills, develop student counseling centers with an integrated socio-academic approach, and launch a periodic social health monitoring system for students. In general, it is very important to develop comprehensive and multifaceted programs that include social well-being, motivation, and academic factors.

Therefore, identifying causes and developing explanatory models should be the main goals of future research. In this regard, suggestions for future research can be categorized and presented in four categories: A: Studies in the field of finding the cause of the lack of correlation between variables include qualitative studies to discover mediating factors affecting the relationship between academic motivation and academic achievement and studies investigating the role of moderator variables such as learning styles, family support, emotional intelligence, study skills, and the level of satisfaction with the university. B: Designing longitudinal studies to investigate the effect of psychological and social interventions such as coping skills training, stress management, and strengthening self-efficacy on social well-being, academic motivation, and academic achievement of students. C: Investigating factors affecting students’ social well-being, such as social interactions, family, university environment, and supportive policies, to provide more targeted solutions. And D: Cross-cultural studies to compare these patterns in universities with different cultural contexts to better explain the relationships between social well-being, motivation, and academic achievement of students.

## Limitations

This study has certain limitations, just like any other research. The cross-sectional design of this study is its primary limitation, as it limits the ability to draw causal inference. In other words, this approach is unable to investigate changes over time or demonstrate a cause-and-effect relationship. Although it might prove a correlation between variables, it is unable to prove that one variable caused the change in another variable. Additionally, unmeasured confounders, such as financial stress, may influence the results, limiting causal inferences. Therefore, it is recommended that future studies, including longitudinal or experimental approaches, establish a causal relationship and consider unmeasured confounder factors. Due to time, resource, and practical constraints, a longitudinal or experimental study design was not feasible for us in this study. A second potential limitation of this study is the heterogeneity within strata, which may introduce variability not fully accounted for in our sampling design. Although we addressed this through covariate adjustment in regression analyses, residual confounding may remain. Future studies with larger samples could consider finer stratification by field of study to improve precision. A third potential limitation of this study is its power to detect small but meaningful effects, particularly those associated with correlation coefficients below 0.2. While we justified our threshold based on prior literature, the ability to identify subtler effects may be limited. Future research with larger sample sizes is recommended to explore these smaller effects more comprehensively. The study’s fourth limitation refers to the findings’ generalizability due to the sample being limited to a specific culture. Because the research sample consisted of Iranian students, the findings may not be generalizable to other cultures and require further investigation. Therefore, to examine the generalizability of the results, it is suggested that the study be repeated in different cultures. The fifth limitation of this study was the lack of direct measurement of SDT components (basic needs of autonomy, competence, and relatedness) as mediating variables in the analysis of the relationship between social well-being and academic motivation, which limited the possibility of a more detailed examination of the mechanisms of influence. Therefore, it is suggested that future studies examine an integrated model that tests the basic needs of SDT as mediating mechanisms between the dimensions of social well-being and academic motivation. Although the use of self-reported GPA poses a potential limitation due to recall or social desirability bias, we sought to mitigate this concern by verifying the reported data against official academic records. The modest variance explained by the regression models is a key limitation. It directly suggests that social well-being, while a significant factor, is one of many contributors to academic motivation and achievement, and a substantial portion of the variance is attributable to unmeasured variables.

## Conclusion

Based on this study, the levels of social well-being and academic motivation among students at DUMS were found to be moderate. Social well-being and its components are significantly correlated, but moderately and weakly with students’ academic motivation. However, no significant direct correlation was found between social well-being and academic achievement. Nonetheless, social well-being and its components are associated with 28.5% and 11.1% of the variance in academic motivation and achievement, respectively. Overall, social well-being, particularly the components of social participation, social solidarity, and social acceptance, is a significant and contributing factor in students’ academic motivation because, through social participation and social acceptance, students develop a greater sense of self-worth. Given the moderate levels of social well-being and academic motivation among students, DUMS managers can help to enhance students’ academic motivation through proper and systematic planning to ensure their social well-being, resulting in improved academic achievement.

## Supplementary Information

Additional file 1.

## Data Availability

The datasets used and analyzed during this research are accessible upon reasonable request from the corresponding author.

## Abbreviations

DUMS: Dezful University of Medical Sciences
SDT: Self-Determination theory
SWBS: Social Well-being Scale
AMS: Academic Motivation Scale
GPA: Grade Point Average
SD: Standard Deviation
VIF: Variance inflation factor
