# Supplementary material for: The impact of social well-being on students’ academic motivation and academic achievement: a case study from Iran
Source: BMC Med Educ. 2025 Nov 14;25:1598. doi: 10.1186/s12909-025-08109-3 (PMC12619280; doi:10.1186/s12909-025-08109-3)
Supplement: Supplementary file 1 — Additional file 1. [file 12909_2025_8109_MOESM1_ESM.docx]

**Table S1. Average score of social well-being, motivation, and educational achievement** **based on demographic variables**

| Variables | | **Social well-being** | | | **Academic motivation** | | | **Academic achievement** | | |
| --- | --- | --- | --- | --- | --- | --- | --- | --- | --- | --- |
|  |  | Average | SD | P-value | Average | SD | P-value | Average | SD | P-value |
| Gender | Female | 75.8 | 11 | 0.09 | 129.5 | 24.8 | 0.035 | 17.3 | 1.2 | <0.001 |
|  | Male | 77.9 | 11.9 |  | 123.1 | 25.9 |  | 16.7 | 1.2 |  |
| Marital status | Single | 76.6 | 11.3 | 0.98 | 127.9 | 25.3 | 0.119 | 17.1 | 1.2 | 0.425 |
|  | Married | 76.9 | 12.3 |  | 119.4 | 24.9 |  | 17.1 | 1 |  |
| Age | 18-22 | 77.2 | 11.3 | 0.321 | 134 | 22.6 | <0.001 | 17.2 | 1.3 | 0.223 |
|  | 23-27 | 75.5 | 11.4 |  | 116.6 | 25.5 |  | 17 | 1.1 |  |
|  | 28-32 | 79.1 | 11 |  | 124.4 | 31.9 |  | 16.8 | 0.8 |  |
| Semesters passed | 1-4 | 77.8 | 1.04 | 0.013 | 136.9 | 2.11 | <0.001 | 16.9 | 0.12 | 0.002 |
|  | 5-8 | 77.75 | 1.3 |  | 122.3 | 2.98 |  | 17.6 | 0.13 |  |
|  | 9-12 | 72.2 | 1.6 |  | 116 | 3.41 |  | 16.8 | 0.21 |  |
|  | 13-16 | 72.5 | 2.1 |  | 107.2 | 3.87 |  | 17.1 | 0.15 |  |
|  | 17-21 | 73.5 | 5.5 |  | 158.5 | 10.5 |  | 16.58 | 1.14 |  |
| Schools | Medical | 74.6 | 10.7 | 0.011 | 127.1 | 24.6 | 0.925 | 16.8 | 1.2 | <0.001 |
|  | Nursing | 78 | 11.7 |  | 126.6 | 27.5 |  | 17.3 | 1.1 |  |
|  | Paramedical | 79.4 | 11.6 |  | 128.1 | 24.2 |  | 17.6 | 1.3 |  |
| Academic level | General medical | 73.8 | 10.2 | 0.001 | 126.1 | 24.4 | 0.257 | 16.7 | 1.2 | <0.001 |
|  | Bachelor | 79.1 | 11.9 |  | 127.4 | 26.1 |  | 17.5 | 1.1 |  |
|  | Associate | 80.7 | 10.4 |  | 143.8 | 25.8 |  | 16.3 | 0.8 |  |
| Field of study | medical | 73.9 | 10.3 | 0.004 | 126.5 | 24.7 | 0.94 | 16.8 | 1.2 | <0.001 |
|  | Public health | 89.6 | 12.7 |  | 140.8 | 18.7 |  | 17.4 | 1.2 |  |
|  | nursing | 78.2 | 12.3 |  | 126.5 | 27.2 |  | 17.4 | 1 |  |
|  | Medical emergencies | 79.2 | 9.8 |  | 131.1 | 34.3 |  | 16.3 | 0.8 |  |
|  | operating room | 80.7 | 10.7 |  | 127.4 | 30.2 |  | 17.3 | 1.2 |  |
|  | anesthesia | 75.5 | 13.5 |  | 129.6 | 16.1 |  | 18.1 | 1 |  |
|  | Laboratory science | 80.8 | 9 |  | 127.2 | 23 |  | 17.6 | 1.4 |  |
